# Renal hemodynamic effects differ between antidiabetic combination strategies: randomized controlled clinical trial comparing empagliflozin/linagliptin with metformin/insulin glargine

**DOI:** 10.1186/s12933-021-01358-8

**Published:** 2021-09-04

**Authors:** Christian Ott, Susanne Jung, Manuel Korn, Dennis Kannenkeril, Agnes Bosch, Julie Kolwelter, Kristina Striepe, Peter Bramlage, Mario Schiffer, Roland E. Schmieder

**Affiliations:** 1 Department of Nephrology and Hypertension, Friedrich-Alexander University Erlangen-Nürnberg, Ulmenweg 18, 91054 Erlangen, Germany; 2 Department of Nephrology and Hypertension, Paracelsus Medical University, Nuremberg, Germany; 3 Department of Cardiology, Friedrich-Alexander University Erlangen-Nürnberg, Erlangen, Germany; 4 Institute for Pharmacology and Preventive Medicine, Cloppenburg, Germany

**Keywords:** Hemodynamics, Intraglomerular, Renal, Type 2 diabetes

## Abstract

**Background:**

Type 2 diabetes causes cardio-renal complications and is treated with different combination therapies. The renal hemodynamics profile of such combination therapies has not been evaluated in detail.

**Methods:**

Patients (N = 97) with type 2 diabetes were randomized to receive either empagliflozin and linagliptin (E+L group) or metformin and insulin glargine (M+I group) for 3 months. Renal hemodynamics were assessed with para-aminohippuric acid and inulin for renal plasma flow (RPF) and glomerular filtration rate (GFR). Intraglomerular hemodynamics were calculated according the Gomez´ model.

**Results:**

Treatment with E+L reduced GFR (p = 0.003), but RPF remained unchanged (p = 0.536). In contrast, M+I not only reduced GFR (p = 0.001), but also resulted in a significant reduction of RPF (p < 0.001). Renal vascular resistance (RVR) decreased with E+L treatment (p = 0.001) but increased with M+I treatment (p = 0.001). The changes in RPF and RVR were different between the two groups (both p_adjust_ < 0.001). Analysis of intraglomerular hemodynamics revealed that E+L did not change resistance of afferent arteriole (R_A_) (p = 0.116), but diminished resistance of efferent arterioles (R_E_) (p = 0.001). In M+I group R_A_ was increased (p = 0.006) and R_E_ remained unchanged (p = 0.538). The effects on R_A_ (p_adjust_ < 0.05) and on R_E_ (p_adjust_ < 0.05) differed between the groups.

**Conclusions:**

In patients with type 2 diabetes and preserved renal function treatment with M+I resulted in reduction of renal perfusion and increase in vascular resistance, in contrast to treatment with E+I that preserved renal perfusion and reduced vascular resistance. Moreover, different underlying effects on the resistance vessels have been estimated according to the Gomez model, with M+I increasing R_A_ and E+L predominantly decreasing R_E_, which is in contrast to the proposed sodium-glucose cotransporter 2 inhibitor effects.

*Trial registration*: The study was registered at www.clinicaltrials.gov (NCT02752113) on April 26, 2016

## Background

Worldwide prevalence of type 2 diabetes is increasing [1]. Combination of metformin and insulin is considered a valid and well established combination therapy in type 2 diabetes [2]. However, according to the updated Position Statement of the American Diabetes Association and European Association for the Study of Diabetes, dual and even triple oral antidiabetic combination therapy is recommended [3]. Among the goals of treatment for type 2 diabetes is the delay of cardiovascular and renal complications, including heart failure, one of the most common and serious complications experienced by about 20–40% of patients with diabetes [4]. There exists an intricate relationship between cardiac and renal function, leading to a vicious and selfperpetuating cycle of diabetes, heart failure and renal insufficiency.

For several decades, after the introduction of renin-angiotensin system (RAS)-blockers [5, 6], no other renoprotective treatment strategy has emerged that exerted, in addition to its cardioprotective effects, renoprotective effects beyond blood glucose and blood pressure control. In 2015, the EMPA-REG OUTCOME trial has triggered great enthusiasm. In patients with type 2 diabetes treated with the sodium-glucose cotransporter 2 (SGLT2) inhibitor empagliflozin, cardiovascular outcome and death as well as hospitalization for heart failure decreased [7] and the progression of kidney disease (as defined by incident or worsening of nephropathy) was attenuated [8].

Previously, several meta-analyses showed beneficial effects of SGLT2 inhibition in type 2 diabetes over a spectrum of cardiovascular and renal risk [9–11].

Noteworthy, in patients treated with empagliflozin an initial drop in estimated glomerular filtration rate (eGFR) occurred, followed by a stabilization of eGFR decline compared to placebo during long-term treatment [8].

The initial decline in eGFR likely reflects attenuation of single-nephron hyperfiltration, which is associated with subsequent long-term renal function preservation [12]. Indeed, data from experimental rodent models [13] and from patients with type 1 diabetes and hyperfiltration [14] support this view. However, corresponding data in type 2 diabetes, which represent 90% of patients with diabetic nephropathy, were not available for a long time and remain scare [15]. Likewise, little is known on the renal and intraglomerular hemodynamic effects of insulin. Again, the nephroprotective effects of glucose lowering therapy with insulin therapy has been shown only in type 1 diabetes [16]. The precise nephroprotective mechanisms remain unclear. In addition, in type 2 diabetes no effect was found on secondary outcomes (e.g. insulin regimens did not affect renal failure or doubling of serum creatinine level), compared to placebo or diet alone [17].

The primary goal of the current study was to assess renal and vascular changes in type 2 diabetes. Findings on vascular structure and function have been published [18]. Here we report the results of the primary renal objectives, namely the renal (GFR, renal plasma flow [RPF] filtration fraction [FF], renal blood flow [RBF] and renal vascular resistance [RVR]) and glomerular hemodynamic effects (intraglomerular pressure [P_glom_] and resistances of the afferent [R_A_] and efferent [R_E_] arterioles) of two antidiabetic combination strategies, an oral combination of empagliflozin with linagliptin and the combination of metformin with insulin glargine in patients with type 2 diabetes.

## Methods

### Study design

This prospective, randomized, controlled, parallel-arm, interventional, open-label, single center study was conducted between April 2016 and November 2018. Participants were recruited from referring physicians and by advertisements in local newspapers in the area of Erlangen-Nürnberg, Germany. Eligible subjects were enrolled consecutively and written informed consent was obtained prior to study inclusion.

Patients entered a run-in phase of 4 weeks if already on stable metformin medication (either 850 mg or 1000 mg bid) for at least 2 months. Patients on two antidiabetic drugs (any kind was accepted), on monotherapy other than metformin or on oral metformin 500 mg bid only rolled over to monotherapy with metformin 850 mg or 1000 mg bid for 3 months, depending on glycated hemoglobin (HbA1c).

After 3 months on metformin monotherapy, baseline assessments of renal and glomerular hemodynamics were performed and immediately thereafter patients were consecutively randomized (1:1) either to empagliflozin/linagliptin (E+L) or metformin/insulin glargine (M+I) combination therapy according to a randomization list provided by the sponsor.

Patients allocated to the E+L group stopped their metformin medication and received empagliflozin 10 mg and linagliptin 5 mg orally once daily. After 14 days empagliflozin was up-titrated to 25 mg (once daily), if fasting blood glucose was ≥ 100 mg/dl and no hypoglycemic symptoms were recognized.

Patients randomized to the M+I group were maintained on their metformin dosage (850 or 1000 mg orally bid) and insulin glargine once daily subcutaneous was added. Initially 2–4 U insulin glargine daily (depending on body weight) was given, and adjusted every third day (telephone counseling) by adding 2 U if fasting blood glucose was not ≤ 125 mg/dl.

The study protocol was approved by the Local Ethics Committee (University of Erlangen-Nürnberg, Germany) and the study was conducted in accordance with the Declaration of Helsinki and the principles of “good clinical practice” guidelines. The study was registered at www.clinicaltrials.gov (NCT02752113).

The financial supporter Boehringer-Ingelheim did not contribute to study conduction, data collection and interpretation of the data.

### Study population

Inclusion criteria were male and female patients with type 2 diabetes aged between 18 and 75 years, HbA1c had to be either ≥ 6.5% (48 mmol/mol) for those on antidiabetic monotherapy or ≥ 6.0% (42 mmol/mol) for those being on dual antidiabetic therapy. Main exclusion criteria were use of insulin, glitazones, dipeptidyl peptidase (DPP)-4 inhibitor or SGLT2 inhibitor within the past 2 months prior randomization. Furthermore, use of loop diuretics was not allowed. Patients with HbA1c > 10.5% (91 mmol/mol) or fasting plasma glucose > 240 mg/dl, urinary albumin to creatinine ratio (UACR) > 300 mg/g creatinine, eGFR < 60 ml/min/1.73 m^2^, and cardio- and cerebrovascular event within the previous 6 months were excluded. Female patients had to have a negative pregnancy test before and during the study period.

### Determination of renal hemodynamics

Renal hemodynamics were determined using the constant-infusion input-clearance technique with inulin (Inutest, Fresenius, Linz, Austria) and sodium p-aminohippurate (PAH) (Daiichi Sankyo, Tokyo, Japan) for GFR and RPF, respectively, (www.crc-erlangen.de) [19, 20]. Briefly, after bolus infusion of inulin and PAH over 15 min and a subsequent constant infusion over 105 min, a steady state between input and renal excretion of the tracer substances is reached. Two blood samples (5 min apart) were collected for the assessment of RPF and GFR at the end of the infusion period [21]. PAH was measured according to previously described methods [22]. Inulin was measured indirectly by converting inulin to fructose and subsequently measuring fructose by an enzymatic method (Boehringer Mannheim, Mannheim, Germany). Each blood sample was measured in duplicate with a coefficient variation of < 5%.

After an official warning due to anaphylactic reactions observed during constant-infusion input-clearance examination with inulin in France, we immediately stopped the application of inulin in our lab (03/2018). As a consequence, we have GFR determined by inulin at baseline as well as after 12 weeks of treatment in only 34 patients of E+L and 31 patients of M+I group, respectively.

FF was calculated as GFR/RPF, RBF as RPF/(1 − haematocrit) and RVR by dividing mean arterial pressure (MAP) with RBF.

### Calculation of intraglomerular hemodynamics

Based on the model originally established by Gomez [23], discussed by Guidi et al. [24] and repeatedly applied in previous studies [25–27], calculation of P_glom_ and R_A_ and R_E_ were performed.

### Statistical analyses

Normal distribution of data was confirmed by Kolmogorov–Smirnov tests before further analysis. Normally distributed data were compared by paired and unpaired student t-tests and are expressed as mean ± standard deviation in text and tables and mean ± standard error of the mean in figures. Since fasting plasma glucose as well as HbA1c was significantly different between treatment arms (E+L vs. M+I), analysis of covariance with HbA1c (p_adjust_) was applied taken this finding (related to the multiple adjustment of insulin glargine dosage, see above) into account. Two-tailed values of p < 0.05 were considered statistically significant. Statistical analyses were performed using IBM SPSS Statistics for Windows, Version 21.0 (IBM Corp., Armonk, NY, USA).

## Results

Baseline clinical characteristics of study population (N = 51 in E+L group and N = 46 in M+I group, respectively) are presented in Table 1.

**Table 1:** Baseline clinical characteristics of study population

Parameter	E+L group	M+I group	p-value
Age (years)	59.9 ± 9.8	60.7 ± 8.9	0.648
Diabetes duration (years)	7.2 ± 5.6	9.0 ± 5.3	0.092
RAS-blockade (−)	26 (51.0%)	27 (58.7%)	0.297
Weight (kg)	90.2 ± 14	93.1 ± 17	0.348
BMI (kg/m^2^)	30.4 ± 3.6	31.3 ± 3.9	0.222
Fasting plasma glucose (mg/dl)	156.2 ± 29	164.7 ± 34	0.240
HbA1c (%/mmol/mol)	7.69 ± 0.7/61 ± 7.7	7.73 ± 0.8/61 ± 8.7	0.780
Office systolic BP (mmHg)	132 ± 13	131 ± 13	0.774
Office diastolic BP (mmHg)	80 ± 8	78 ± 9	0.489
Office heart rate (beats/min)	71.3 ± 10	72.2 ± 10	0.671

Data are means ± SD or n (%)

RAS: renin-angiotensin system; BP: blood pressure

Glycemic parameters like fasting plasma glucose (E+L: 157.3 ± 29.5 vs. 136.4 ± 23.8 mg/dl, p < 0.001; M+I: 164.0 ± 34.5 vs. 122.0 ± 20.0 mg/dl, p < 0.001) and HbA1c (E+L: 7.7 ± 0.7 (61 ± 7.7) vs. 7.3 ± 0.8% (56 ± 8.7 mmol/mol), p < 0.001; M+I: 7.8 ± 0.8 (62 ± 8.7) vs. 7.0 ± 0.7% (53 ± 7.7 mmol/mol), p < 0.001) were significantly reduced after 12 weeks in both treatment groups, but to a greater extent with M+I compared to E+L treatment (e.g. HbA1c − 0.8 ± 0.57 (− 8.7 ± 6.2) vs. − 0.4 ± 0.7% (− 4.4 ± 7.7 mmol/mol), p = 0.001). In contrast, only E+L treatment resulted in a reduction of weight (89.8 ± 14.4 vs. 87.3 ± 13.5 kg, p < 0.001) and body mass index (BMI) (30.2 ± 3.5. vs. 29.4 ± 3.2 kg/m^2^, p < 0.001), both being unchanged in M+I group (weight: 93.8 ± 16.1 vs. 94.1 ± 16.2 kg, p = 0.234; BMI: 31.4 ± 3.9 vs. 31.5 ± 3.9 kg/m^2^, p = 0.257), resulting in a significant difference in favour for the E+L treatment (weight: − 2.5 ± 2.5 vs. 0.3 ± 1.6 kg, p_adjust_ < 0.001; BMI: − 0.8 ± 0.8 vs. 0.1 ± 0.5 kg/m^2^, p_adjust_ < 0.001) after 12 weeks between the groups.

Compared to baseline, hematocrit increased significantly in E+L group (41.1 ± 3.1 vs. 44.5 ± 3.3%, p < 0.001), whereas hematocrit remained unchanged in M+I group (41.3 ± 2.9 vs. 41.8 ± 3.6%, p = 0.109), resulting in a significant difference between the groups (p_adjust_ < 0.001). There was a significant increase in total protein in E+L group (6.29 ± 3.7 vs. 6.48 ± 3.3 g/dl, p < 0.001), whereas no change was observed in M+I group (6.34 ± 3.4 vs. 6.38 ± 3.8 g/dl, p = 0.229), leading to a significant difference between treatment group (p_adjust_ = 0.019).

In comparison to baseline, there was a significant reduction of MAP only in E+L group (93.4 ± 8.6 vs. 90.3 ± 8.0 mmHg, p = 0.009), whereas MAP remained unchanged in M+I group (92.5 ± 7.2 vs. 93.4 ± 6.8 mmHg, p = 0.264), resulting in a significant difference after 12 weeks of treatment between the groups (p_adjust_ = 0.021).

### Renal hemodynamics

Compared to baseline, RPF remained unchanged whereas RBF (p = 0.021) increased after 12 weeks of E+L treatment. Measured GFR (p = 0.003) was reduced after 12 weeks of treatment with E+L treatment, whereas FF was maintained (p = 0.151). Treatment with E + L effected a lower RVR (p = 0.001) after 12 weeks.

**Fig. 1:**
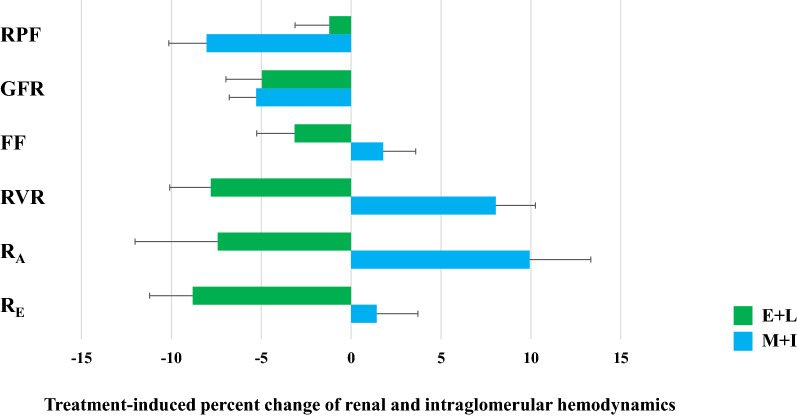
Treatment-induced percent change of renal and intraglomerular hemodynamics

Combination therapy with M+I lead to a reduction of RPF (p < 0.001) and RBF (p = 0.002) compared to baseline. Measured GFR (p = 0.001) was reduced after 12 weeks of M+I treatment compared to baseline and FF was maintained (p = 0.337). Of note, we observed an increment of RVR (p = 0.001) compared to baseline values in the M+I group after 12 weeks (Table 2, Fig. 1).

**Table 2:** Renal and intraglomerular hemodynamics

	E+L group				M + I group				E+L vs. M+I
Parameter	Baseline	12 weeks	Δ	p-value	Baseline	12 weeks	Δ	p-value	p_adjust_-value
RPF (ml/min)^*^	623 ± 114	615 ± 115	− 7.6 ± 86	0.536	653 ± 150	600 ± 121	− 52 ± 94	< 0.001	0.041
GFR (ml/min)^†^	127 ± 13	120 ± 14	− 6.3 ± 12	0.003	127 ± 15	120 ± 13	− 6.7 ± 10	0.001	0.899
FF (%)^†^	21.6 ± 3.1	20.9 ± 3.0	− 0.7 ± 2.7	0.151	21.2 ± 2.8	21.5 ± 3.2	0.38 ± 2.2	0.337	0.200
RBF (ml/min)^*^	1061 ± 199	1113 ± 220	53 ± 157	0.021	1112 ± 249	1036 ± 217	− 76 ± 159	0.002	< 0.001
RVR (mmHg)^*^	91 ± 20	84 ± 18	− 7 ± 15	0.001	87 ± 21	94 ± 21	7 ± 13	0.001	< 0.001
P_glom_ (mmHg)^†^	61.6 ± 3.2	61.1 ± 3.4	− 0.46 ± 3.1	0.387	61.7 ± 3.1	60.9 ± 3.2	− 0.86 ± 2.6	0.073	0.661
R_A_ (dyn*s/cm^5^)^†^	2547 ± 880	2358 ± 734	− 189 ± 685	0.116	2579 ± 798	2835 ± 800	256 ± 485	0.006	0.023
R_E_ (dyn*s/cm^5^)^†^	2361 ± 407	2152 ± 389	− 208 ± 330	0.001	2316 ± 351	2349 ± 435	33 ± 298	0.538	0.011
R_E_/R_A_ (−)^†^	1.02 ± 0.36	1.01 ± 0.37	− 0.01 ± 0.4	0.836	0.96 ± 0.25	0.87 ± 0.2	− 0.09 ± 0.17	0.004	0.597
MAP (mmHg)	93.4 ± 8.6	90.3 ± 8.0	− 3.1 ± 8.0	0.009	92.5 ± 7.2	93.4 ± 6.8	1.0 ± 5.9	0.264	0.021

Dara are means ± SD

^*^ N = 50 (E+L group) and N = 46 (M+I group), respectively

^†^N = 34 (E+L group) and N = 31 (M+I group), respectively

A comparison of the two combination therapies disclosed a different renal hemodynamic pattern treatment: E+L treatment maintained RPF and RBF, whereas M+I treatment effected a reduction in RPF (p_adjust_ = 0.041) and RBF (p_adjust_ < 0.001). Similar changes on GFR and FF were observed in both treatment groups. After 12 weeks of treatment, RVR was decreased after E+L treatment and increased in M+I group, resulting in a significant difference between both treatment arms (p_adjust_ < 0.001) (Table 2, Fig. 1).

### Intraglomerular hemodynamics

After 12 weeks of E+L treatment, estimation of intraglomerular hemodynamic parameters revealed no changes of P_glom_ (p = 0.387) and of R_A_ (p = 0.116), but a significant reduction of R_E_ (p = 0.001). Treatment with M+I resulted in no change of P_glom_ (p = 0.073), a significant reduction of R_A_ (p = 0.006), but no change in R_E_ (p = 0.538) compared to baseline.

Comparing both combination therapies, we observed disparate effects on intraglomerular hemodynamics, namely a predominant reduction at postglomerular site (R_E_) after E+L treatment and predominant increment of resistance at the preglomerular site (R_A_) after M+I treatment, (R_A_: − 189 ± 685 vs. 256 ± 485 dyn*s/cm^5^, p_adjust_ = 0.023; R_E_: − 208 ± 330 vs. 33 ± 298 dyn*s/cm^5^, p_adjust_ = 0.011) between the treatment groups (Table 2, Fig. 1).

## Discussion

Applying the gold-standard technique for the assessment of renal hemodynamics, namely constant-infusion input-clearance technique with inulin and PAH, we found that treatment with E+L maintained RPF, increased RBF and reduced RVR. In contrast, treatment with M+I reduced RPF and RBF and increased RVR after 12 weeks of medication. Thus, our data disclosed a significantly different renal hemodynamic profile between the two treatment combinations.

With advancing kidney disease, renal perfusion decreases and pharmacological further reduction of RPF and RBF, or conversely increase of RVR must be regarded as undesirable side effects. Here, we report for the first time that insulin induces an increment of RVR and a reduction of glomerular blood supply in type 2 diabetes. Animal experiments using microperfused arterioles have reported conflicting results. Data showing that insulin predominately dilates afferent arterioles [28] as well as data demonstrating vasoconstriction of afferent arteriole with physiological concentrations of insulin have been published [29]. The discrepant results may be related to different timing of insulin administration. By applying the Gomez model to the baseline and treatment data, treatment with insulin resulted in an increment of resistance at the afferent preglomerular site (R_A_). These findings found in the group randomized to M+I therapy after 3 months seems to be attributed to the insulin treatment, since all patients were on at least 3 months metformin monotherapy at baseline and metformin dosage was kept stable throughout the 12 weeks of additional insulin medication.

Our data are in line with the findings of the EMPA-REG OUTCOME trial, showing a reduction of eGFR immediately after initiating SGLT2 inhibition [8]. Such a decrease has been commonly related to the tubuloglomerular feedback (TGF). An increased sodium concentration in the tubular system close to the macula densa, a consequence of SGLT2 inhibition, increases arteriolar tone at the afferent preglomerular site and thereby decreases GFR. Previously, in animal model of type 1 diabetes a single dose of the SGLT2 inhibitor empagliflozin resulted in an increment of afferent arteriolar tone, and hence reduction of glomerular hyperfiltration, considered the first step stage of diabetic nephropathy. Moreover, subsequent experiments revealed that adenosine signaling through TGF is the key pathophysiological pathway [30].

Support for this view how SGLT2-inhibitors exert nephroprotective effects come from studies in type 1 diabetes. It was found that short-term therapy with empagliflozin of 8 weeks resulted in a reduction of GFR in hyperfiltrating patients, whereas GFR remained unchanged in normofiltrating patients [14]. In addition, a post-hoc analyses revealed that hyperfiltrating patients with type 1 diabetes have significantly lower R_A_, indicative of afferent vasodilation, but unaltered R_E_ compared to normofiltrating patients with type 1 diabetes. Moreover, empagliflozin treatment resulted in an increment of R_A_ towards levels of normofiltrating ones in hyperfiltrating patients with type 1 diabetes with no effect on R_E_ [31].

Although, there is no general established the cut-off of glomerular hyperfiltration, studies assessing renal- and intraglomerular hemodynamics defined a GFR ≥ 135 ml/min/1.73 m^2^ as hyperfiltration. Therefore, our patients as well as at least most patients of the EMPA-REG OUTCOME trial [7] (placebo: 73.8 ± 21.1 vs. pooled empagliflozin: 74.2 ± 21.6 ml/min/1.73 m^2^, p = ns), albeit not based on inulin-measured GFR seems to be not in this range.

It is noteworthy, to mention that in the postulated classic course of diabetic kidney disease two normal filtration phases can be encountered (flanking probably a period of hyperfiltration), one at 100% of nephron mass and one at approximately 50% of nephron mass [4]. Based on an autopsy study of type 1 diabetes and type 2 diabetes it was postulated that diabetic kidney lesions may develop before the onset of clinical findings [32]. Albeit single-nephrons are at a hyperfilrating stage, overall GFR can be normal (or even slightly reduced) due to functional nephron loss. Therefore, it has been suggested that FF may reflect hyperfiltration at the single-nephron level more precisely [4]. However, in accordance to our data with respect to intraglomerular hemodynamics (see above), there was only a numerical reduction of FF after combined E+L treatment, without reaching significance or showing a clear trend-level, hence not supporting a modulation of presumed hyperfiltration status at single-nephron level.

Therefore, the second main finding of our study in patients with type 2 diabetes is in contrast to these reports. When applying the Gomez formula, no significant impact on R_A_, but significantly diminished R_E_ was observed after 3 months of therapy in the E+L group. We attribute these intraglomerular changes in resistances to empaglifozin treatment, since in a former randomized, cross-over study we found that 4-weeks of linagliptin monotherapy exerts no alterations on renal and intraglomerular hemodynamics, including estimates of the afferent and efferent resistances, compared to placebo [27]. This is in accordance with available literature showing that in patients with type 2 diabetes sitagliptin did not affect RPF and GFR as well as intrarenal hemodynamics, namely R_A_ and R_E_ with the exception of a modestly reduced P_glom_. However, it is stated that validity and clinical relevance of the slight sitagliptin-induced P_glom_ reduction remains speculative [33]. Moreover, in another study from the same group linagliptin (compared to glimepiride) did not affect GFR, RPF as well as intrarenal hemodynamics (including P_glom_) [34], which is in accordance with our own previous study [27].

Clearly, the obvious explanation of these discrepant results of SGLT2-inhibition on intraglomerular hemodynamics is related to different study populations and likely also different stages of kidney function. We examined older patients with type 2 diabetes with an eGFR > 60 ml/min/1.73 m^2^, whereas Cherney et al. derived their conclusions from data obtained in (younger) patients with type 1 diabetes [31] and experimental models of diabetic nephropathy in type 1 diabetes [30].

Notably, our data are in accordance with the recently published RED trial by Bommel et al. demonstrating that dapagliflozin treatment resulted in reduction of GFR due to postglomerular vasodilation rather than preglomerular vasoconstriction in patients with type 2 diabetes [15]. Therefore, we suggest that modulation of intraglomerular hemodynamics due to SGLT2 inhibition may substantially differ whether applied in type 1 diabetes or type 2 diabetes.

This study has several limitations. First, the human renal microcirculation cannot be visualized/examined in vivo directly, and the used Gomez model is based on some assumptions [23]. However, comparison of intrarenal hemodynamics within an individual, and within a short period of time are considered to be reliable in humans. This model appeared to be in particular applicable in our patients with type 2 diabetes who had no macroalbuminuria and eGFR ≥ 60 ml/min/1.73 m^2^. In an excellent review, Bjornstad et al. recommended obtaining accurate measurements of GFR and RBF to limit the signal-to-noise ration with Gomez´ equations. By doing so, they conclude that application of Gomez´ equations, provide specific and accurate data on R_A_ and R_E_ [35]. Second, there was a significant difference in glycemic control (e.g. HbA1c), which was not prespecified in the study protocol, but we have applied additional statistical analyses to take this finding into account. Moreover, analyses of the EMPA-REG OUTCOME trial disclose that glycemic control is not the key mechanism of beneficial effects of SGLT-2 inhibition [36]. Addressing other proposed mechanism of the beneficial effects of SGLT2 inhibition, like modulating inflammation and fibrosis [37, 38], is beyond the scope of our single-center pilot study. Third, patients were partly on concomitant medication (e.g. RAS blockade), that may alter intrarenal hemodynamics. However, concomitant medication was kept stable throughout the study. Fourth, data cannot be extrapolated to type 2 diabetes in general (e.g. hyperfiltrating patients) as well as to type 1 diabetes or patients with reduced eGFR presumably having hyperfiltration on a single nephron level. Nevertheless, our findings may have important implications, since normofiltrating patients with type 2 diabetes represents by far most diabetic patients suffering on diabetes.

## Conclusions

Taken together, two antidiabetic combination strategies revealed a completely disparate hemodynamic pattern in the kidney. We found that insulin on top of metformin therapy targets predominantly preglomerular site and increases renal vascular resistance, thereby diminishing renal blood supply, and second that SGLT2-inhibition with empagliflozin impacts predominantly on postglomerular site, decreases renal vascular resistance and preserves renal perfusion in patients with type 2 diabetes and an preserved renal function. Since there exists an intricate relationship between cardiac and renal function, leading to a vicious and selfperpetuating cycle of heart failure and renal insufficiency, our results may explain the cardiac effects of antidiabetic agents beyond their renal effects.

## Data Availability

The datasets used and/or analysed during the current study are available from the corresponding author on reasonable request.

## Abbreviations

BMI: Body mass index
DPP: Dipeptidyl peptidase
E: Empagliflozin
(e)GFR: (Estimated) glomerular filtration rate
FF: Filtration fraction
HbA1c: Glycated hemoglobin
I: Insulin glargine
L: Linagliptin
M: Metformin
MAP: Mean arterial pressure
PAH: p-Aminohippurate
P_glom_: Intraglomerular pressure
R_A_: Resistance of the afferent arterioles
RBF: Renal blood flow
R_E_: Resistance of the efferent arterioles
RPF: Renal plasma flow
RVR: Renal vascular resistance
SGLT2: Sodium-glucose cotransporter 2
UACR: Urinary albumin to creatinine ratio
